# Integrating Body Schema and Body Image in Neurorehabilitation: Where Do We Stand and What’s Next?

**DOI:** 10.3390/brainsci15040373

**Published:** 2025-04-03

**Authors:** Rocco Salvatore Calabrò

**Affiliations:** IRCCS Centro Neurolesi “Bonino-Pulejo”, Cda Casazza, SS 113, 98123 Messina, Italy; roccos.calabro@irccsme.it

Given the widespread debate surrounding the definitions and functional roles of “Body Schema” and “Body Image”, these constructs have become central to understanding motor control and rehabilitation, particularly for individuals with neurological impairments [1]. Although often used interchangeably, body schema and body image represent distinct cognitive and sensorimotor phenomena with unique contributions to movement execution and perception. Body schema is an unconscious, sensorimotor representation that is crucial for spatial coordination and movement automation, while body image refers to a conscious, cognitive-perceptual representation that influences self-awareness and emotional states [2]. This distinction is particularly important in the context of rehabilitation, as the specific contributions of each representation can inform targeted therapeutic interventions. The growing number of neurological disorders that affect motor function, especially those impairing the upper limbs, underscores the need for a paradigm shift in rehabilitation strategies, in order to integrate both body schema and body image to optimize recovery [2].

Traditional rehabilitation approaches have predominantly focused on restoring muscle strength and joint mobility. However, contemporary research, as emphasized by Sattin et al. in their cutting-edge narrative review, suggests that interventions targeting both body schema and body image can significantly enhance motor recovery [3]. The authors highlight that understanding the role of these body representations is crucial for addressing motor deficits and developing effective rehabilitation techniques. In their work, they examine the different definitions and models of body schema and body image, and explore empirical settings used to test these theories, particularly focusing on interventions for upper limb impairments. They underscore the need for a new phenomenological approach to rehabilitation that places body schema at the forefront as a fundamental and intrinsic component of action in space. By better understanding these representations, rehabilitation efforts can be more effectively tailored to the needs of patients.

Recent studies have demonstrated the importance of sensorimotor retraining, virtual reality therapy, and brain–computer interfaces in recalibrating body schema to improve movement execution [4]. These innovative interventions engage body schema through immersive techniques, such as virtual reality, which provides patients with real-time, interactive environments that facilitate sensorimotor integration and recovery. Additionally, the mirror neuron system (MNS) has emerged as a crucial neurophysiological mechanism underpinning motor rehabilitation. Techniques such as action observation and motor imagery leverage MNS activation to facilitate motor learning, even in the absence of voluntary movement [4,5]. The role of the MNS in driving neural plasticity and motor recovery has been emphasized by Calabrò et al. [6], particularly in patients with severe motor impairments. The activation of the MNS during observed or imagined movements enhances motor pathway engagement, effectively priming the brain for movement execution. This neurophysiological framework provides a powerful avenue for rehabilitation, allowing patients to strengthen motor circuits and improve functional outcomes even when direct movement is not yet possible.

A particularly promising approach in neurorehabilitation is Action Observation Treatment (AOT), which involves patients watching goal-directed actions with the intention of replicating them. This technique has been shown to improve motor performance and foster neural reorganization in stroke patients who struggle with motor execution [7]. The subsequent practice of motor imagery (MI)**,** or the mental simulation of movement, further strengthens the neural connections involved in movement planning and execution. By mentally rehearsing actions after observing them, patients can reinforce the motor pathways involved, even in the absence of physical movement. This combination of action observation followed by motor imagery provides a powerful mechanism for motor recovery, activating the same neural circuits used during actual movement, thus promoting neuroplasticity and improving motor function [8].

The integration of these techniques with neurophysiological methods like transcranial magnetic stimulation (TMS) and functional electrical stimulation (FES) offers additional avenues for enhancing motor recovery. TMS modulates cortical excitability, promoting neuroplasticity and facilitating motor learning [9]. FES, on the other hand, can provide external stimulation to impaired muscles, helping bridge the gap between neural intention and physical execution [10]. These approaches, combined with interventions targeting body schema and body image, are particularly beneficial for patients with severe motor impairments.

Moreover, Sattin’s work highlights the importance of addressing the psychological aspects of rehabilitation. The relationship between body image disturbances and psychological conditions like depression and anxiety as well as eating disorders is well-documented, particularly in individuals recovering from neurological injuries [11]. A negative body image can hinder recovery and reduce a patient’s engagement in therapy. Thus, integrating psychological interventions such as cognitive-behavioral therapy and mindfulness-based approaches alongside motor rehabilitation can foster a more positive body image, which ultimately supports motor recovery and enhances therapeutic outcomes [11].

The role of body schema in neurorehabilitation is crucial in conditions that disrupt spatial coordination and motor execution, such as stroke, traumatic brain injury, and spinal cord injury [2]. In these disorders, damage to brain regions responsible for processing body schema can impair movement accuracy and hinder body awareness. Rehabilitative techniques that recalibrate body schema, such as virtual reality, action observation, and motor imagery, help patients regain spatial awareness and improve voluntary movement quality [2,7,8]. A comprehensive rehabilitation approach must address both body schema and body image to ensure holistic recovery. Interventions targeting the body schema focus on restoring automatic motor functions through repetitive task practice and sensory integration, whereas therapies aimed at body image work to reshape the individual’s conscious perception of their body, often employing cognitive-behavioral techniques to improve self-awareness and emotional well-being. Body schema-based interventions are particularly effective for patients with hemiplegia, dysphagia, and motor deficits, as the integration of body schema is essential for restoring functional motor behavior and fostering independence [2]. Furthermore, conditions like hemispatial neglect and somatoagnosia, which affect body awareness and spatial attention, must be appropriately addressed to support recovery and improve the patient’s interaction with their environment. These combined efforts, targeting both unconscious motor functions and conscious body perceptions, offer the best potential for meaningful rehabilitation outcomes [12].

The rise in artificial intelligence (AI) and machine learning in rehabilitation is another promising development. AI-driven systems can analyze movement patterns and adapt therapy in real-time, offering personalized rehabilitation programs that are more responsive to individual patient needs. These technologies, in combination with wearable sensors and neurofeedback devices, ensure precise monitoring of motor function and rehabilitation progress, thereby optimizing recovery outcomes [13]. By tailoring interventions based on patient-specific data, AI can significantly improve the efficiency and effectiveness of rehabilitation programs.

The integration of these emerging technologies, combined with psychological and sensorimotor rehabilitation approaches, holds immense potential for improving patient outcomes. As Sattin’s review suggests, the interdisciplinary collaboration between neuroscientists, clinicians, and engineers is essential for developing innovative rehabilitation solutions [4]. By refining our understanding of body schema and body image, and leveraging these novel technologies, we can create more effective, patient-centered rehabilitation strategies that improve the quality of life for individuals with motor impairments.

In conclusion, integrating body schema and body image into neurorehabilitation represents a pivotal advancement in treatment strategies for neurological disorders. By addressing both sensorimotor and cognitive dimensions of recovery, rehabilitation can become more comprehensive and tailored to individual needs. The work by Sattin et al. not only deepens our theoretical understanding of body representations but also highlights the translational potential of this knowledge for developing evidence-based interventions. Future research should focus on refining assessment tools to better distinguish between impairments in body schema and body image, allowing for more targeted therapies. Additionally, there is a need to establish standardized, yet adaptable, rehabilitation protocols that can be personalized based on factors such as lesion location, neuroplastic potential, and individual cognitive profiles. Emerging technologies, including virtual reality, neurofeedback, and brain–computer interfaces, should be further explored to enhance rehabilitation outcomes by providing immersive and interactive training environments. Longitudinal studies are also essential to assess the durability of interventions and their impact on long-term functional recovery. By bridging theoretical insights with innovative clinical applications, future research can significantly advance neurorehabilitation, ultimately improving quality of life for individuals with neurological disorders.
